# Clinical presentations, diagnostics, treatments and treatment costs of children and adults with febrile illness in a tertiary referral hospital in south-eastern Guinea: A retrospective longitudinal cohort study

**DOI:** 10.1371/journal.pone.0262084

**Published:** 2022-01-10

**Authors:** Manuel Raab, Lisa M. Pfadenhauer, Dansira Doumbouya, Guenter Froeschl

**Affiliations:** 1 Division of Infectious Diseases and Tropical Medicine, University Hospital (LMU), Munich, Germany; 2 Institute of Medical Informatics, Biometry and Epidemiology, Pettenkofer School of Public Health, Ludwig Maximilian University Munich, Munich, Germany; 3 Paediatric Service, Hôpital Régional de Nzérékoré, Nzérékoré, Guinea; Kaohsuing Medical University Hospital, TAIWAN

## Abstract

**Background:**

Febrile illness is frequent among patients in the tropics. It is caused by a wide variety of common diseases such as malaria or gastrointestinal infections but also by less common but highly contagious pathogens with epidemic potential. This study describes the clinical features of adult and paediatric patients with febrile illness in in the largest tertiary referral hospital in south-eastern Guinea, a region at high risk for viral haemorrhagic fever outbreaks. The study further compares their diagnostic characteristics, treatments and outcomes with non-febrile patients in order to contribute to the local epidemiology of febrile illness.

**Methods:**

We used retrospective data collection to record demographic and clinical data of all incoming patients during a study period of three months. For the follow-up study of inpatients, we retrospectively reviewed patient charts for diagnostic characteristics, diagnoses and outcomes.

**Results:**

Of the 4317 incoming patients during the study period, 9.5% had a febrile illness. The most used diagnostic measures to identify causative agents in febrile patients were point-of-care tests and most treatments relied on antibiotics. Most common discharge diagnoses for febrile inpatients were malaria (9.6% adults, 56.7% children), salmonella gastroenteritis/typhoid (10.6% adults, 7.8% children) and respiratory infection/pneumonia (5.3% adults, 18.7% children). Inpatient mortality for children was significantly higher in febrile than non-febrile children (18.5% vs. 5.1%, p<0.001) and considerably higher in febrile than non-febrile adults (29.8% vs. 25.0%, p = 0.404).

**Conclusions:**

Malaria, respiratory infection and gastroenteritis are considered the main causes for febrile illness. The wide reliance on rapid diagnostic tests to diagnose febrile patients not only risks to over- or under-diagnose certain diseases but also leaves the possibility of highly infectious diseases in febrile patients unexplored. Furthermore, the heavy reliance on antibiotics risks to cause antimicrobial resistance. High mortality rates in febrile patients, especially children, should be of concern to public health authorities.

## Background

Fever is a common reason for patients to seek healthcare in the tropics [1–3]. It is usually associated with non-specific gastrointestinal or respiratory symptoms but may also present itself as an isolated symptom. Fever is often used synonymously with the term “febrile illness”, which is defined as an illness with an elevated body temperature of at least 38.0°C or higher [4]. Possible causes of febrile illness include a wide spectrum of pathogens such as bacterial bloodstream infections (e.g. *Salmonella entertica* subtypes), mycobacterial bloodstream infections (e.g. *Mycobacterium tuberculosis*), bacterial zoonosis (e.g. brucellosis), protozoal infections (e.g. African trypanosomiasis), fungal infections (e.g. cryptococcus) and viral infections (e.g. rhinoviruses) [5]. In malaria endemic regions, malaria is often the default diagnosis for febrile illness, partly because of its high prevalence and partly because of the predominance of malaria eradication programs in the past decade which led to the over-diagnosing of malaria [6–8]. Especially in low-resource healthcare settings, identifying non-malarial febrile illness can be challenging due to impaired diagnostic capacities and few point-of care tests for most microorganisms [9]. While adequate treatment of febrile illness ideally depends on the identification of the causative agent, febrile illness in low-resource healthcare settings is usually treated with calculated antimicrobial and/or antimalarial medications, increasing the risk of drug resistance [10].

Next to more common causes of febrile illness in the tropics, certain pathogens with epidemic potential have emerged as equally important causes for febrile illness [11]. Notably, the 2014–2016 West African Ebola virus outbreak as well as the recent 2021 Ebola virus outbreak in south-eastern Guinea highlighted the importance of pathogen-specific screening and surveillance in West Africa for potentially infectious patients presenting with fever–fever being one of the most common symptoms amongst patients infected with Ebola virus [12, 13]. Guinea–one of the poorest African nations with a fragile healthcare system–was amongst the most affected countries by the Ebola virus epidemic [14]. Particularly its south-eastern region is now considered at high risk for outbreaks of viral haemorrhagic fevers such as Ebola virus disease, Marburg virus disease, Lassa fever and Crimean-Congo haemorrhagic fever [15–20]. All of these highly contagious diseases are known to cause febrile illness in patients at an early stage of infection [21–24]. But also other potentially fatal infectious diseases causing febrile illness such as meningitis, leptospirosis or dengue have been reported in the region [25–29].

In this study we describe epidemiological, clinical and diagnostic characteristics, current treatment strategies, and outcomes of adult and paediatric patients with febrile illness treated at the largest referral hospital in south-eastern Guinea. We further compare these clinical features of febrile patients with non-febrile patients. Our study provides a profile of a typical Guinean provincial referral hospital by highlighting its procedures and capacities in regard to diagnosing and treating febrile illness. Thereby, we hope to contribute to a better understanding of the local epidemiology of febrile illness in a region at risk for infectious disease outbreaks. We point to further management needs in Guinean healthcare structures regarding febrile illness amongst patients.

## Methods

### Study setting

Our study was carried out at the Hôpital Régional de N’zérékoré (N’zérékoré Regional Hospital, HRNZ), a tertiary provincial referral hospital in south-eastern Guinea. N’zérékoré is Guinea’s second largest city with more than 300,000 inhabitants and is the capital of Guinea’s forested region, also known as *Guinée Forestière* with a population of over two million [30]. Bordering Sierra Leone, Liberia and Ivory Coast, the region has a tropical climate with a wet season lasting approximately from May until November and the dry season from December until April. *Guinée Forestière* is in south-eastern Guinea and is known as the region where the 2014–2016 West African Ebola outbreak most likely started [31]. A new Ebola outbreak close to N’zérékoré city was declared in early 2021 and several cases were hospitalized in the HRNZ prior to being laboratory confirmed [32].

With a capacity of approximately 175 beds and services in Internal Medicine, Surgery, Intensive Care, Gynaecology/Obstetrics, Ophthalmology and Dental Care, the HRNZ is amongst the three largest hospitals in the country. It is the largest referral hospital in *Guinée Forestière* receiving emergency patients as well as patients requiring specialized care from surrounding urban and rural areas. The hospital laboratory is equipped for basic serological, haematological, bacteriological and clinical biochemistry testing. Furthermore, an additional laboratory for viral haemorrhagic fevers was established in 2014. Supply shortages may, however, constrain laboratory diagnostic capacities. The only possibilities for imaging purposes are one X-ray and one ultrasound machine. An overview of the most relevant diagnostic tests for infectious diseases can be found under in S1 Table.

Upon hospital-entry, triage staff records main symptoms and body temperatures of all non-emergency patients in order to orient patients to the hospital’s different outpatient services. Severely ill patients are immediately directed to the emergency room. At the outpatient services, physicians may decide that patients require inpatient care and will send them to the emergency room for further examinations and inpatient admission. Patients requiring obstetric care or seeking access to the specialized HIV clinic located within the hospital compound may bypass hospital triage. Thus, all inpatients except HIV clinic and obstetric patients are handled at the level of the emergency room.

The emergency room separates paediatric, adult medical and surgical patients. Paediatric surgical patients are treated in the adult surgical ward. Physicians examine patients, administer mRDT if necessary and initiate first treatments. They further decide whether patients require inpatient care or not.

Patient records are kept in various registers at hospital triage and the emergency room while patient charts are compiled only for inpatients at the level of the emergency room.

### Data sources and study-design

Using a retrospective study design, we analysed data of all in- and outpatients who came to the HRNZ between December 2, 2018 and March 1, 2019 (dry season), except obstetric and HIV clinic patients. Details on study design and data sources are well-explained elsewhere [33]. Data was extracted from hospital records and patient charts at three levels. First, triage data for all incoming patients comprised socio-demographic characteristics, symptoms, measured body temperatures and referral service. Second, we recorded diagnostic results, treatments, hospitalization status, suspected diagnoses (the most important three) and outcomes for all adult and paediatric emergency patients, except surgical patients, deceased patients during emergency treatment and patients whose body temperatures had not been recorded; these patients were excluded. Third, laboratory diagnostics, primary discharge diagnoses and outcomes of adult and paediatric medical inpatients were extracted from patient charts by the authors MR (adults) and DD (children) after patients were discharged and patient charts completed at the end of each week. We transferred and coded the above hand-written information from patient charts into digital line lists and reviewed each other’s data transfer and results. When differences were discovered, MR and DD reviewed the weekly patient charts together to come to a common conclusion. When patient charts were incomplete or unclear, we consulted the responsible healthcare worker for missing information.

For this study, we stratified patient data of all incoming patients (in- and outpatients) by measured axillary body temperatures (cut-off 38.0°C). In our data, medical adult and paediatric emergency patients are subdivided into two groups (without fever: < 38.0°C and with fever: ≥ 38.0°C). They are described and compared by the variables age, symptoms, suspected diagnosis, diagnostics, initial treatment and admission status (inpatient vs. outpatient). Febrile and non-febrile patients requiring inpatient care are further compared to add more details regarding laboratory diagnostics, discharge diagnoses and outcomes.

Next to the above, we calculated an approximation for the average inpatient healthcare costs per adult and per paediatric inpatient based on laboratory costs, costs for medications and hospitalization costs per patient. Laboratory costs were calculated based on hospital price lists and all laboratory and imaging diagnostics performed on inpatients. Costs for medications were calculated based on the average amount of medications prescribed per inpatient and the average price per medication as indicated by the hospital pharmacy. We excluded the ten most expensive, yet rarely used medications from our approximation to reduce risk of skewed over-estimating inpatient medication costs. Hospitalization costs were calculated based on price lists as provided by the hospital administration.

### Data analysis

Patient symptoms and diagnoses were coded according to the International Classification of Primary Care, 2^nd^ edition (ICPC-2). All data was recorded and coded with Microsoft Excel 16 and analysed using IBM SPSS 25. Descriptive statistics were generated and proportions were compared using Pearson’s-Chi² Test and Exact Fisher Test. Statistical significance was determined at p ≤ 0.05. Because of extreme outliers in age, we used non-parametric median and interquartile range (IQR) to describe the age of all incoming patients.

### Ethical considerations

Ethical approval for this study was granted by the Guinean Ethics Committee for Research in Health (opinion number 103/CNERS/18) and the Ethics Committee for Medical Research at the Ludwig-Maximilians-Universität (LMU), Munich, Germany (opinion number 18–834). Before its implementation, the study was presented to the regional health authorities and the HRNZ directorate who both consented to its implementation. Since data was collected as part of routine clinical practice and for the purpose of this retrospective study extracted in an anonymized fashion, and in the further analysis presented in an aggregate manner, no informed consent was asked from patients.

## Results

### General characteristics of all incoming patients

**Fig 1:**
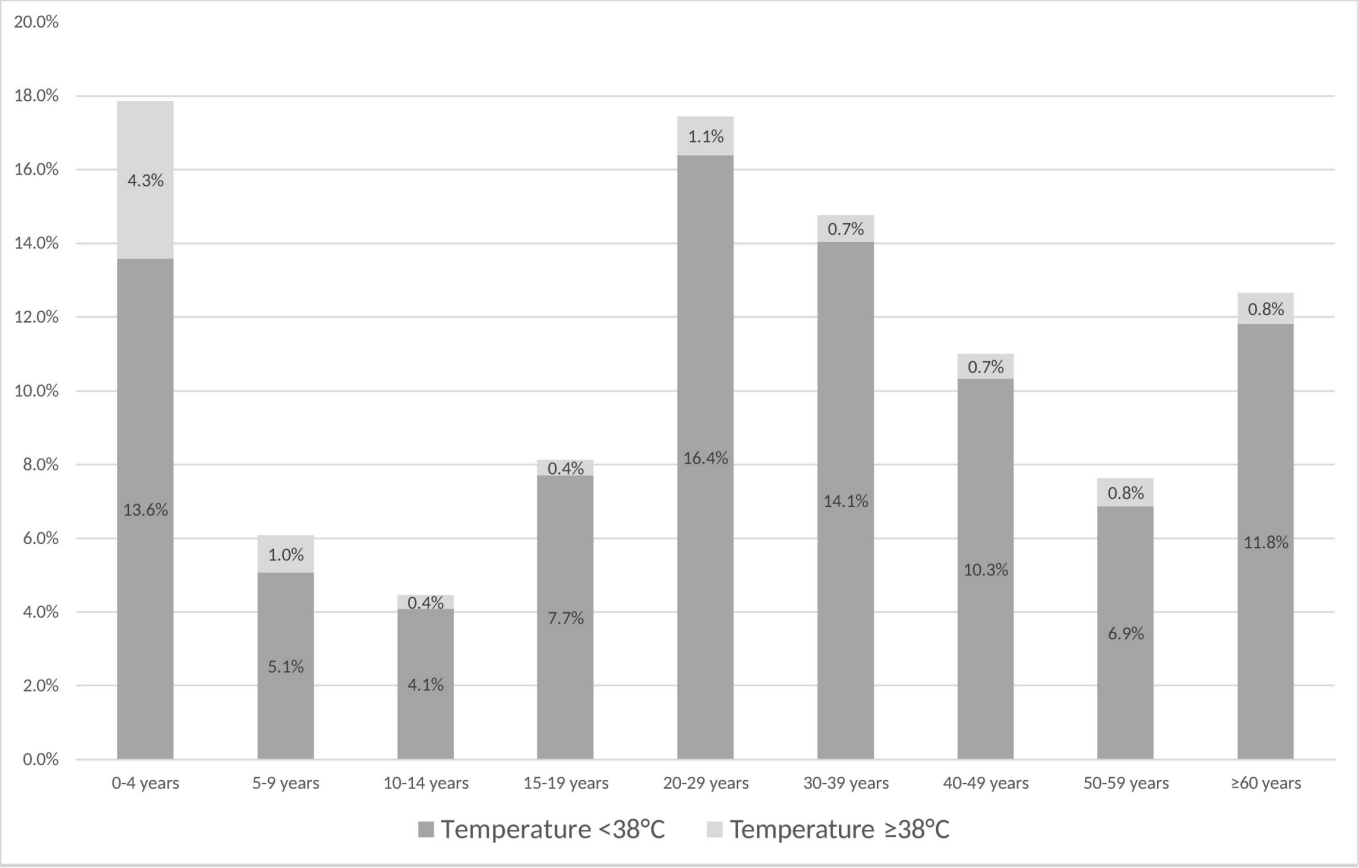
Proportions of non-febrile and febrile patients by age group.

4317 patients were registered as in- and outpatients during our study period. Of those patients, 2616 patients (60.6%) were handled by triage, 1178 patients (27.3%) by the adult emergency room and 523 patients (12.1%) by the paediatric emergency room (Table 1). In total, 67 patients (1.6%) deceased in the emergency room during treatment, of which 56 in the adult emergency room and 11 in the paediatric emergency room. Almost half of all the patients were male (2121/4317; 49.1%), the other half female (2193/4317; 50.8%). The majority of all patients (3375/4317; 78.2%) came from urban areas, the rest (795/4317; 18.4%) from rural areas. While patients reported fever as the most frequent primary reason for seeking care at the hospital, none of the 2616 patients handled by triage had a measured axillary body temperature ≥38,0°C (febrile). However, with regard to the emergency facilities, 15.4% (182/1178) of adult emergency patients and 43.8% (229/523) of paediatric emergency patients resulted to be febrile, amounting to a total 9.5% (411/4317) of all incoming patients being febrile upon hospital entry. For 274 patients (6.3%) no body temperature was recorded. Of all patients, most febrile patients were within the age group 0–4 years (4.3%; 174/4043; Fig 1). The three most frequent referral services were internal medicine (35.4%), paediatrics (20.2%) and surgery (14.5%). Deceased patients, surgical patients and patients where body temperature was not recorded were excluded from the following analysis.

**Table 1:** Characteristics of all incoming patients.

	Total	Triage	Adult emergency room	Pediatric emergency room
Total Number of Patients N	4317	2616	1178	523
**General Characteristics**				
Median Age–years (IQR)	27 (11–45)	29 (16–45)	35 (23–55)	1 (1–4)
Male Sex–n/N (%)	2121/4317 (49.1)	1134/2616 (43.3)	691/1178 (58.7)	296/523 (56.6)
Female Sex–n/N (%)	2193/4317 (50.8)	1482/2616 (56.7)	484/1178 (41.1)	227/523 (43.4)
Sex not registered–n/N (%)	3/4317 (0.1)	0/2616 (0.0)	3/1178 (0.3)	0/523 (0.0)
Residence in urban area–n/N (%)	3375/4317 (78.2)	2127/2616 (81.3)	863/1178 (73.3)	385/523 (73.6)
Residence in rural area–n/N (%)	795/4317 (18.4)	487/2616 (18.6)	183/1178 (15.5)	125/523 (23.9)
Residence not registered–n/N (%)	147/4317 (3.4)	2/2616 (0.1)	132/1178 (11.2)	13/523 (2.5)
**Referred Service**				
Internal Medicine–n/N (%)	1527/4317 (35.4)	840/2616 (32.1)	687/1178 (58.3)	0/523 (0.0)
Pediatrics–n/N (%)	872/4317 (20.2)	360/2616 (13.8)	0/1178 (0.0)	512/523 (97.9)
Surgery–n/N (%)	625/4317 (14.5)	190/2616 (7.3)	435/1178 (36.9)	0/523 (0.0)
Ophthalmology–n/N (%)	446/4317 (10.3)	446/2616 (17.0)	0/1178 (0.0)	0/523 (0.0)
Gynecology–n/N (%)	353/4317 (8.2)	353/2616 (13.5)	0/1178 (0.0)	0/523 (0.0)
Dental Clinic–n/N (%)	220/4317 (5.1)	220/2616 (8.4)	0/1178 (0.0)	0/523 (0.0)
Ear Nose Throat (ENT) Clinic–n/N (%)	182/4317 (4.2)	182/2616 (7.0)	0/1178 (0.0)	0/523 (0.0)
Not recorded–n/N (%)	25/4317 (0.6)	25/2616 (1.0)	0/1178 (0.0)	0/523 (0.0)
Deceased during treatment–n/N (%)	67/4317 (1.6)	0/2616 (0.0)	56/1178 (4.8)	11/523 (2.1)
**Body Temperature**				
<38°C–n/N (%)	3632/4317 (84.1)	2585/2616 (98.8)	781/1178 (66.3)	266/523 (50.9)
≥38°C–n/N (%)	411/4317 (9.5)	0/2616 (0.0)	182/1178 (15.4)	229/523 (43.8)
Not recorded–n/N (%)	274/4317 (6.3)	31/2616 (1.2)	215/1178 (18.2)	28/523 (5.4)
**Most frequent primary reasons for consultation**	Fever, Musculoskeletal injury, Eye problems	Eye problems, Fever, Chest pain	Musculoskeletal injury, Abdominal pain, Head injury	Fever, Asthenia, Cough

### Clinical features of medical adult and paediatric emergency patients: Febrile vs. non-febrile patients

Of 1119 medical adult and paediatric emergency patients whose body temperatures were recorded, 34.9% (391/1119) were febrile, with the larger proportion of febrile patients being paediatric patients (26.1% adults vs. 46.3% children; Table 2). Overall, asthenia (52.6%; 589/1119), loss of appetite (36.9%; 413/1119), vomiting (30.8%; 345/1119) and cough (30.3%; 339/1119) were the most frequently reported symptoms amongst medical emergency patients. Adult febrile patients were more likely than non-febrile patients to report asthenia (50.0% vs 36.1%, p = 0.002), diarrhoea (17.7% vs 10.1%, p = 0.020) and cough (25.6% vs. 11.0%, p<0.001). They were also more likely to receive a mRDT upon hospital entry (65.9% vs. 49.2%, p<0.001). In total, 45 adult patients (7.2%; 45/629) had a positive mRDT result, of which 19 patients were febrile and 26 patients non-febrile (11.6% vs. 5.6%, p = 0.014). For children, none of the reported symptoms were more specific for febrile in comparison to non-febrile patients. MRDTs were performed on all but one paediatric patient with results being almost equally distributed between febrile and non-febrile children: 50.0% of all children (47.1% febrile vs 52.5% non-febrile, p = 0.277) received a negative mRDT result and 49.8% (52.4% febrile vs. 47.5% non-febrile, p = 0.319) a positive result.

**Table 2:** Clinical features of febrile vs. non-febrile medical adult and paediatric emergency patients.

	Total	Adult medical patients				Pediatric medical patients			
		Total	Febrile N = 164	Non-febrile N = 465	p-value	Total	Febrile N = 227	Non-febrile N = 263	p-value
Total Number of Patients N–n/N (%)	1119	629	164/629 (26.1)	465/629 (73.9)		490	227/490 (46.3)	263/490 (53.7)	
**Most frequent symptoms (besides fever)**									
*General symptoms*									
Asthenia–n/N (%)	589/1119 (52.6)	250/629 (39.7)	82/164 (50.0)	168/465 (36.1)	0.002	339/490 (69.2)	163/227 (71.8)	176/263 (66.9)	0.281
Loss of appetite–n/N (%)	413/1119 (36.9)	158/629 (25.1)	50/164 (30.5)	108/465 (23.2)	0.075	255/490 (52.0)	123/227 (54.2)	132/263 (50.2)	0.415
Headache–n/N (%)	194/1119 (17.3)	171/629 (27.2)	46/164 (28.0)	125/465 (26.9)	0.761	23/490 (4.7)	12/227 (5.3)	11/263 (4.2)	1.000
Dizziness–n/N (%)	113/1119 (10.1)	112/629 (17.8)	33/164 (20.1)	79/465 (17.0)	0.406	1/490 (0.2)	0/227 (0.0)	1/263 (0.4)	1.000
Digestive symptoms									
Vomiting–n/N (%)	345/1119 (30.8)	111/629 (17.6)	35/164 (21.3)	76/465 (16.3)	0.094	234/490 (47.8)	98/227 (43.2)	136/263 (62.0)	0.070
Diarrhea–n/N (%)	224/1119 (20.0)	78/629 (12.4)	29/164 (17.7)	49/465 (10.5)	0.020	146/490 (29.8)	65/227 (28.6)	81/263 (30.8)	0.622
Abdominal pain–n/N (%)	221/1119 (19.7)	176/629 (28.0)	40/164 (24.4)	136/465 (29.2)	0.266	45/490 (9.2)	21/227 (9.3)	24/263 (9.1)	1.000
*Respiratory symptoms*									
Cough–n/N (%)	339/1119 (30.3)	93/629 (14.8)	42/164 (25.6)	51/465 (11.0)	<0.001	246/490 (50.2)	114/227 (50.2)	132/263 (50.2)	1.000
Dyspnoea–n/N (%)	104/1119 (9.3)	72/629 (11.4)	17/164 (10.4)	54/465 (11.6)	0.774	32/490 (6.5)	14/227 (6.2)	18/263 (6.8)	0.855
**Malaria rapid diagnostic test (mRDT)**									
Negative–n/N (%)	537/1119 (48.0)	292/629 (46.4)	89/164 (54.3)	203/465 (43.7)	0.023	245/490 (50.0)	107/227 (47.1)	138/263 (52.5)	0.277
Positive–n/N (%)	289/1119 (25.8)	45/629 (7.2)	19/164 (11.6)	26/465 (5.6)	0.014	244/490 (49.8)	119/227 (52.4)	125/263 (47.5)	0.319
Not performed–n/N (%)	293/1119 (26.2)	292/629 (46.4)	56/164 (34.1)	236/465 (50.8)	<0.001	1/490 (0.2)	1/227 (0.4)	0/263 (0.0)	0.463
**Most frequent suspected diagnosis**									
Malaria–n/N (%)	632/1119 (56.5)	163/629 (25.9)	57/164 (34.8)	106/465 (22.8)	0.002	469/490 (95.7)	221/227 (97.4)	248/263 (94.3)	0.118
Gastroenteritis–n/N (%)	376/1119 (33.6)	285/629 (45.3)	73/164 (44.5)	212/465 (45.6)	0.855	91/490 (18.6)	42/227 (18.5)	49/263 (18.6)	1.000
Pneumonia/Respiratory infection–n/N (%)	356/1119 (31.8)	51/629 (8.1)	22/164 (13.4)	29/465 (6.2)	0.007	305/490 (62.2)	140/227 (61.7)	165/263 (62.7)	0.852
Gastroduodenal Ulcer–n/N (%)	106/1119 (9.5)	106/629 (16.9)	25/164 (15.2)	81/465 (17.4)	0.305	0/490 (0.0)	0/227 (0.0)	0/263 (0.0)	
Stroke–n/N (%)	54/1119 (4.8)	54/629 (8.6)	6/164 (3.7)	48/465 (10.3)	0.009	0/490 (0.0)	0/227 (0.0)	0/263 (0.0)	
**Selected emergency medications**									
*Antibiotics*									
Ampicillin–n/N (%)	650/1119 (58.1)	316/629 (50.2)	89/164 (54.3)	227/465 (48.8)	0.239	334/490 (68.2)	149/227 (65.6)	185/263 (70.3)	0.285
Metronidazole–n/N (%)	189/1119 (16.9)	69/629 (11.0)	18/164 (11.0)	51/465 (11.0)	1.000	120/490 (24.5)	54/227 (23.8)	66/263 (25.1)	0.753
Gentamycin–n/N (%)	103/1119 (9.2)	1/629 (0.2)	1/164 (1.0)	0/465 (0.0)	1.000	102/490 (20.8)	46/227 (20.3)	56/263 (21.3)	0.824
Ceftriaxone–n/N (%)	97/1119 (8.7)	47/629 (7.5)	13/164 (6.7)	34/465 (7.3)	0.863	50/490 (10.2)	23/227 (10.1)	27/263 (10.3)	1.000
Amoxicillin–n/N (%)	22/1119 (2.0)	6/629 (1.0)	1/164 (1.0)	5/465 (1.1)	1.000	16/490 (3.3)	4/227 (1.8)	12/263 (4.6)	0.124
Ciprofloxacin–n/N (%)	8/1119 (0.7)	7/629 (1.1)	2/164 (1.2)	5/465 (1.1)	1.000	1/490 (0.2)	1/227 (0.4)	0/263 (0.0)	0.463
*Antipyretic/Analgesic*									
Paracetamol–n/N (%)	514/1119 (45.9)	350/629 (55.6)	119/164 (72.6)	231/465 (49.7)	<0.001	164/490 (33.5)	123/227 (54.2)	41/263 (15.6)	<0.001
Diclofenac–n/N (%)	26/1119 (2.3)	26/629 (4.1)	3/164 (1.8)	23/465 (4.9)	0.109	0/490 (0.0)	0/227 (0.0)	0/263 (0.0)	
*Antimalarials*									
Artesunate–n/N (%)	231/1119 (20.6)	22/629 (3.5)	11/164 (6.7)	11/465 (2.4)	0.014	209/490 (42.7)	103/227 (45.4)	106/263 (38.0)	0.273
Artemether/Lumefantrine–n/N (%)	28/1119 (2.5)	14/629 (2.2)	3/164 (1.8)	11/465 (2.4)	1.000	14/490 (2.9)	8/227 (3.5)	6/263 (2.3)	0.430
**Hospitalization status**									
Inpatient–n/N (%)	865/1119 (77.3)	418/629 (66.5)	121/164 (73.8)	297/465 (63.9)	0.021	447/490 (91.2)	209/227 (92.1)	238/263 (90.5)	0.632
Outpatient–n/N (%)	254/1119 (22.7)	211/629 (33.5)	43/164 (26.2)	168/465 (36.1)	0.021	43/490 (8.2)	18/227 (7.9)	25/263 (9.5)	0.632

**Fig 2:**
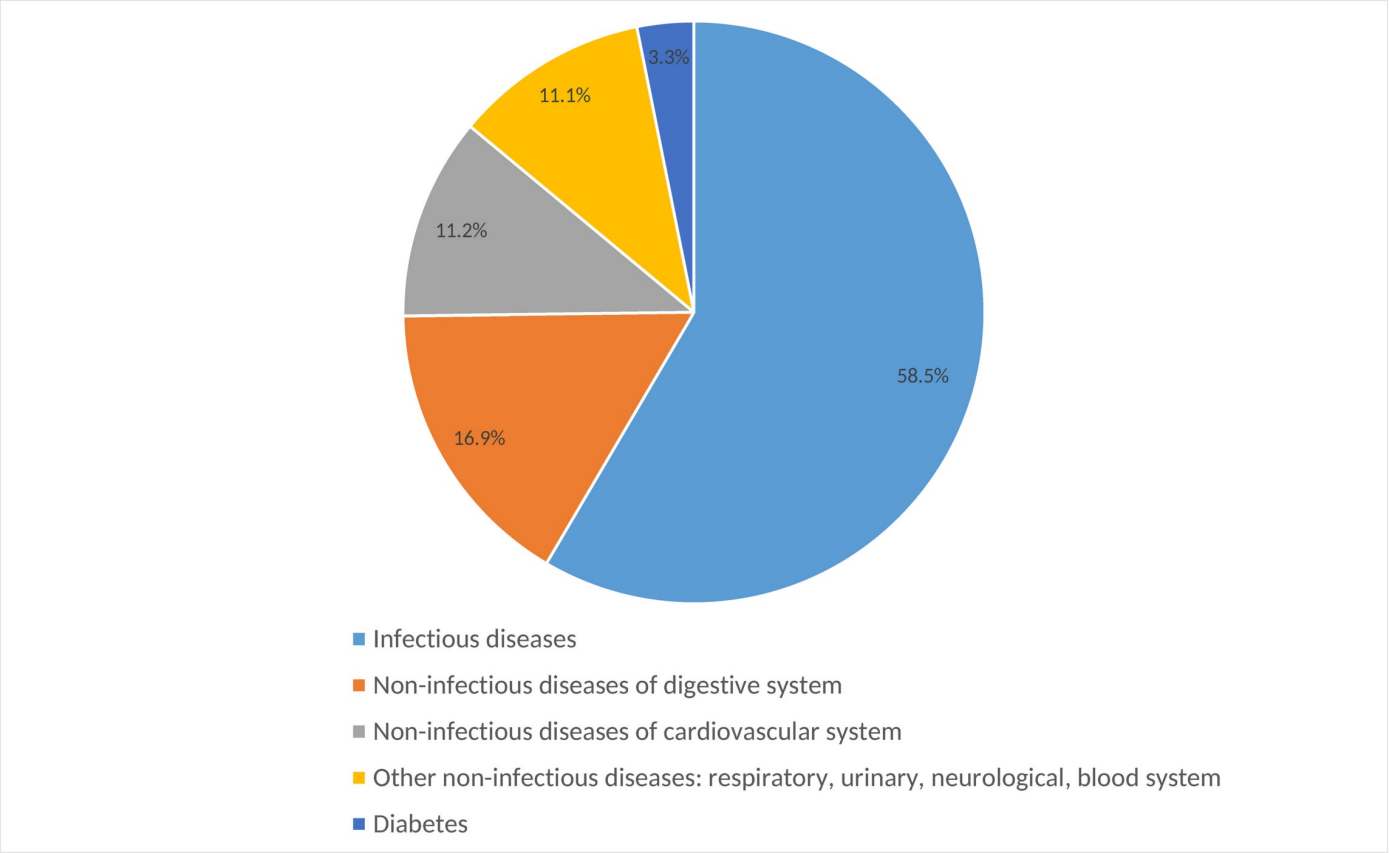
Suspected diagnosis group in medical adult emergency patients.

**Fig 3:**
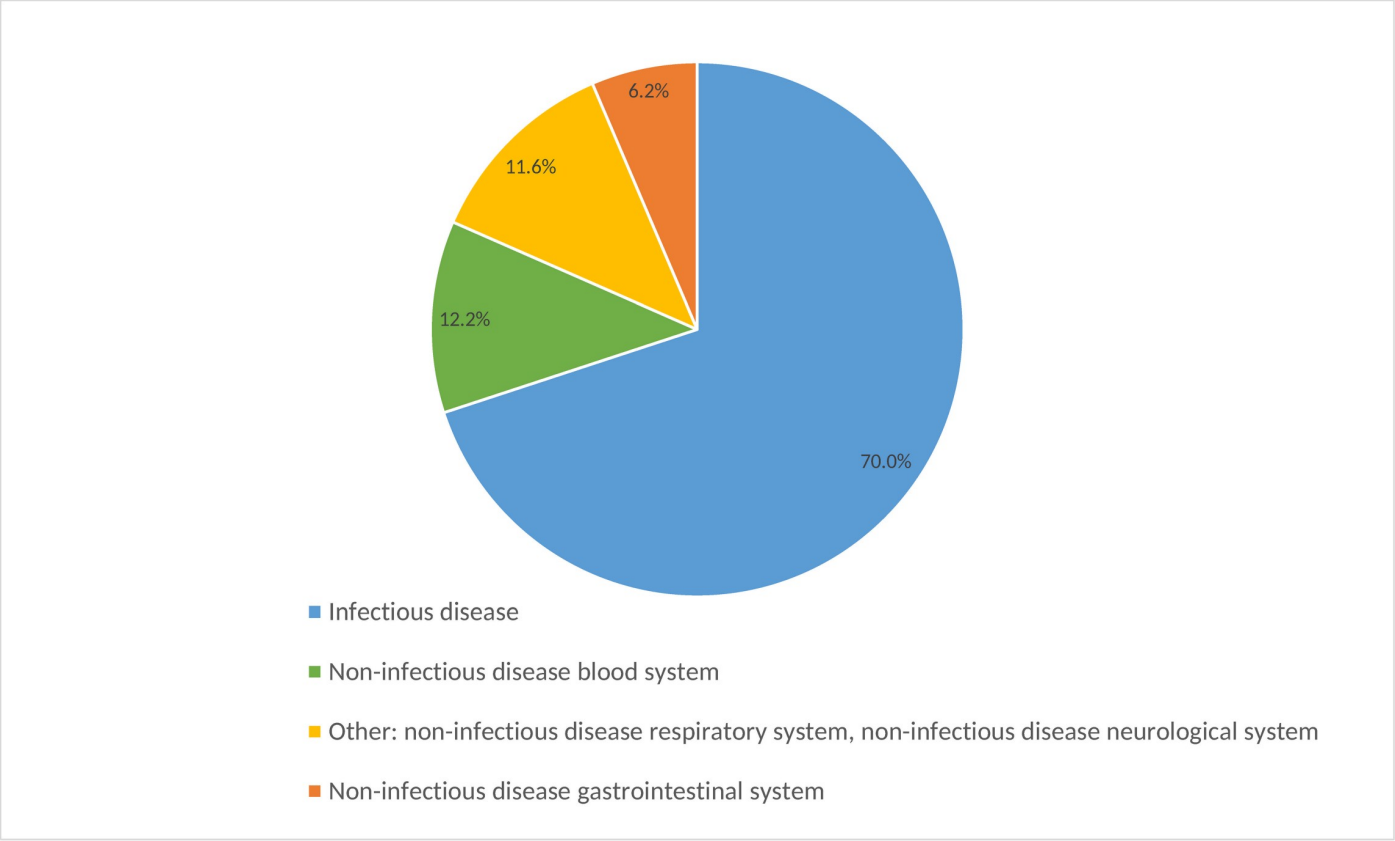
Suspected diagnosis group in medical paediatric emergency patients.

The three most frequently suspected diagnoses as per adult medical emergency patient were salmonella gastroenteritis/typhoid (45.3%; 286/629), malaria (25.9%; 163/629) and gastroduodenal ulcer (16.9%; 106/629). Only malaria was significantly more often diagnosed amongst febrile as compared to non-febrile adults (34.8% vs. 22.8%, p = 0.002). With regard to all diagnoses suspected in adult medical emergency patients combined, 58.5% (594/1016) belonged to the diagnosis group infectious diseases, 16.3% (166/1016) to non-infectious diseases of the gastrointestinal tract and 11.2% (114/1016) to non-infectious cardiovascular diseases (Fig 2). In children, the three most frequently suspected diagnoses were malaria (95.7%; 469/490), respiratory infection/pneumonia (62.2%; 305/490) and gastroenteritis (18.6%; 91/490). There was no significant difference in the proportion of suspected diagnoses between febrile and non-febrile children. In total, 70.0% (892/1275) of all suspected diagnoses combined amongst children in the emergency room belonged to the diagnosis group infectious diseases, 12.2% (156/1275) to non-infectious diseases of the blood system and 6.4% (82/1275) to non-infectious diseases of the gastrointestinal tract (Fig 3).

Treatments for medical emergency patients consisted mainly of antibiotic, analgesic/antipyretic and antimalarial treatment. For adult patients, ampicillin (50.2%; 316/629) and paracetamol (55.6%; 350/629) were by far the most commonly used medications. For children, ampicillin was given to 68.2% (334/490) of patients, artesunate to 42.7% (209/490) of patients and paracetamol to 33.5% (164/490) of patients. For both adults and children metronidazole was another frequently prescribed antibiotic (11.0% adults, 24.5% children). Furthermore, gentamycin was often used for paediatric patients (20.8%; 102/490). Altogether, only paracetamol was significantly more often used in the groups of febrile adult patients (72.6% vs. 49.8%, p<0.001) and febrile paediatric patients (54.2% vs. 15.6%, p<0.001) in comparison to non-febrile patients.

### Diagnostics, discharge diagnoses and outcomes of medical adult and paediatric inpatients: Febrile vs. non-febrile patients

**Fig 4:**
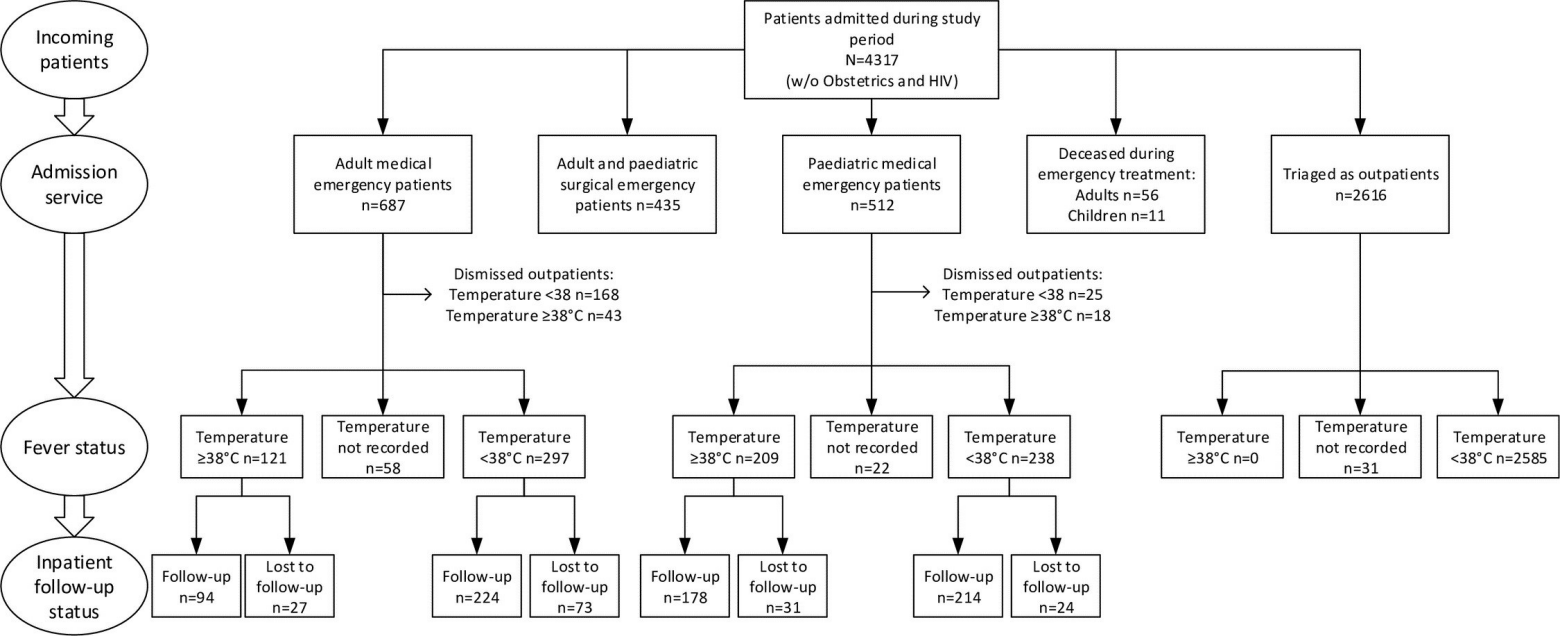
Study flowchart.

Of the 1119 medical emergency patients, 418 adults and 447 children were admitted as inpatients. 100 adult inpatients (23.9%; 100/418) and 55 paediatric inpatients (12.3%; 55/447) were lost to follow-up, leaving 94 febrile (29.6%; 94/318) and 224 non-febrile (70.4%; 224/318) adult inpatient charts and 178 febrile (45.4%; 178/392) and 214 non-febrile (54.6%; 214/392) paediatric inpatient charts for review (Fig 4).

On average, adult febrile inpatients were hospitalized for almost one day longer than non-febrile inpatients (5.9 days vs. 5 days; Table 3). Paediatric inpatients stayed hospitalized less days than adults: 3.5 days on average for febrile children and 3.2 days for non-febrile children. Overall, 56.8% (403/710) of all inpatients were discharged with improved health, 24.3% (172/710) either self-discharged against medical advice or without notifying hospital staff and 18.0% (128/710) deceased in inpatient care. The larger proportion of deceased inpatients was amongst adults (26.4% adults vs. 11.2% children). However, febrile children were significantly more likely to die during hospitalization than non-febrile children (18.5% vs. 5.1%, p<0.001), which only reached a level of a trend for adult inpatients (29.8% vs. 25.0%, p = 0.404). The main causes of death were respiratory infection, malaria and gastroenteritis for children and stroke, gastroenteritis and malaria for adults.

**Table 3:** Diagnostics and outcomes of febrile vs. non-febrile medical adult and paediatric inpatients.

	Total	Adult medical inpatients				Paediatric inpatients			
		Total	Febrile N = 94	Non-febrile N = 224	p-value	Total	Febrile N = 178	Non-febrile N = 214	p-value
Total Number of Patients N–n/N (%)	710	318	94/318 (29.6)	224/318 (70.4)		392	178/392 (45.4)	214/392 (54.6)	
Days hospitalized–Mean in days (SD)	4.3 (3.0)	5.3 (4.0)	5.9 (4.1)	5.0 (3.9)		3.3 (2.0)	3.5 (1.8)	3.2 (2.1)	
**Most frequently performed diagnostics and results**									
*Malaria rapid diagnostic test (mRDT)*									
Positive–n/N (%)	208/710 (29.3)	20/318 (6.3)	6/94 (6.4)	14/224 (8.0)	1.000	188/392 (48.0)	90/178 (50.6)	98/214 (45.8)	0.416
Negative–n/N (%)	350/710 (49.3)	148/318 (46.5)	59/94 (62.8)	89/224 (39.7)	<0.001	202/392 (51.5)	87/178 (48.9)	115/214 (53.7)	0.361
*Thick blood smear (TBS)*									
Positive–n/N (%)	48/710 (6.8)	14/318 (4.4)	4/94 (4.3)	10/224 (4.5)	1.000	34/392 (8.7)	17/178 (9.6)	17/214 (7.9)	0.594
Negative–n/N (%)	224/710 (31.5)	85/318 (26.7)	38/94 (40.4)	47/224 (21.0)	0.001	139/392 (35.5)	59/178 (33.1)	80/214 (37.4)	0.397
*Widal TO/TH*									
Positive–n/N (%)	129/710 (18.2)	53/318 (16.7)	20/94 (21.3)	33/224 (14.7)	0.187	76/392 (19.4)	37/178 (20.8)	39/214 (18.2)	0.608
Negative–n/N (%)	39/710 (5.5)	11/318 (3.5)	4/94 (4.3)	7/224 (3.1)	0.737	28/392 (7.1)	13/178 (7.3)	15/214 (6.5)	1.000
*Cerebrospinal fluid*									
Pathological (indicating bacterial infection)–n/N (%)	11/710 (1.5)	2/318 (0.6)	2/94 (2.1)	0/224 (0.0)	0.087	9/392 (2.3)	5/178 (2.8)	4/214 (1.9)	0.737
Normal–n/N (%)	3/710 (0.4)	0/318 (0.0)	0/94 (0.0)	0/224 (0.0)		3/392 (0.8)	1/178 (0.6)	2/214 (0.9)	1.000
*Stool microscopy parasites*									
Positive–n/N (%)	3/710 (0.4)	2/318 (0.6)	1/94 (1.1)	1/224 (0.4)	0.504	1/392 (0.3)	0/178 (0.0)	1/214 (0.5)	1.000
Negative–n/N (%)	18/710 (2.5)	10/318 (3.1)	2/94 (2.1)	8/224 (3.6)	0.729	8/392 (2.0)	4/178 (2.2)	4/214 (1.9)	1.000
*HIV antibody test*									
Positive–n/N (%)	20/710 (2.8)	20/318 (6.3)	11/94 (11.7)	9/224 (4.0)	0.020	0/392 (0.0)	0/178 (0.0)	0/214 (0.0)	
Negative–n/N (%)	42/710 (5.9)	41/318 (12.9)	16/94 (17.0)	25/224 (11.2)	0.198	1/392 (0.3)	1/178 (0.6)	0/214 (0.0)	0.455
*Syphilis TPHA*									
Positive–n/N (%)	8/710 (1.1)	8/318 (2.5)	2/94 (2.1)	6/224 (2.7)	1.000	0/392 (0.0)	0/178 (0.0)	0/214 (0.0)	
Negative–n/N (%)	26/710 (4.1)	25/318 (7.9)	9/94 (9.6)	16/224 (7.1)	0.496	1/392 (0.3)	1/178 (0.6)	0/214 (0.0)	0.455
*Toxoplasmosis IgG/IgM*									
Positive–n/N (%)	5/710 (0.7)	5/318 (1.6)	2/94 (2.1)	3/224 (1.3)	0.634	0/392 (0.0)	0/178 (0.0)	0/214 (0.0)	
Negative–n/N (%)	4/710 (0.6)	4/318 (1.3)	0/94 (0.0)	4/224 (1.8)	0.323	0/392 (0.0)	0/178 (0.0)	0/214 (0.0)	
*Sputum Tuberculosis*									
Positive–n/N (%)	1/710 (0.1)	1/318 (0.3)	0/94 (0.0)	1/224 (0.4)	1.000	0/392 (0.0)	0/178 (0.0)	0/214 (0.0)	
Negative–n/N (%)	3/710 (0.4)	3/318 (0.9)	1/94 (1.1)	2/224 (0.9)	1.000	0/392 (0.0)	0/178 (0.0)	0/214 (0.0)	
*Hbs Antigen*									
Positive–n/N (%)	7/710 (1.0)	7/318 (2.2)	1/94 (1.1)	6/224 (2.7)	0.678	0/392 (0.0)	0/178 (0.0)	0/214 (0.0)	
Negative–n/N (%)	19/710 (2.7)	19/318 (6.0)	6/94 (6.4)	13/224 (5.8)	0.801	0/392 (0.0)	0/178 (0.0)	0/214 (0.0)	
**Outcomes**									
Improvement of health–n/N (%)	403/710 (56.8)	140/318 (44.0)	43/94 (45.7)	97/224 (43.3)	0.712	263/392 (67.1)	115/178 (64.6)	148/214 (69.2)	0.207
Transferred–n/N (%)	7/710 (1.0)	6/318 (1.9)	1/94 (1.1)	5/224 (2.2)	0.674	1/392 (0.3)	0/178 (0.0)	1/214 (0.5)	1.000
Deceased–n/N (%)	128/710 (18.0)	84/318 (26.4)	28/94 (29.8)	56/224 (25.0)	0.404	44/392 (11.2)	33/178 (18.5)	11/214 (5.1)	<0.001
Self-discharge against medical advice–n/N (%)	85/710 (12.0)	64/318 (20.1)	17/94 (18.1)	47/224 (21.0)	0.646	21/392 (5.3)	10/178 (5.6)	11/214 (5.1)	1.000
Self-discharge without notice–n/N (%)	87/710 (12.3)	24/318 (7.5)	5/94 (5.3)	19/224 (8.5)	0.484	63/392 (16.1)	20/178 (11.2)	43/214 (20.1)	0.019

The most frequent diagnostic tests performed amongst all inpatients were mRDT (78.6%; 558/710), thick blood smear (TBS, 38.3%; 272/710), Widal TO/TH test (23.7%; 168/719) and HIV antibody screening test (8.7%; 62/710), which was mainly used in adult inpatients. Overall, there were no significant differences in the proportion of positive malaria tests between febrile and non-febrile patients. MRDT were positive in 6.3% (20/318) of all adult inpatients (6.6% febrile vs. 8% non-febrile, p = 1.000) and in 48% (188/392) of all paediatric inpatients (50.6% febrile vs. 45.8% non-febrile, p = 0.416). TBS showed presence of *P*. *falciparum* in 4.4% (14/318) of adult inpatients (4.3% febrile vs. 4.5% non-febrile, p = 1.000) and in 8.7% (34/392) of paediatric inpatients (9.6% febrile vs. 7.9% non-febrile, p = 0.594).

Of all inpatients who were tested for malaria by means of mRDT, 67 adult inpatients and 172 paediatric inpatients were also tested for malaria through TBS (Table 4). For adult inpatients, a positive TBS was significantly more likely when the mRDT had been positive: 27.3% (3/11) of adult inpatients with a positive TBS were also tested positive by means of mRDT whereas only 3.6% (2/56) with a negative TBS were tested positive by means of mRDT (2/56, p = 0.006). In other words, 72.7% (8/11) of adult inpatients with a positive TBS received a negative mRDT while 96.4% (54/56) of adult inpatients with a negative TBS also received a negative mRDT result. For children, 5.9% (2/34) of inpatients with a positive TBS were also tested positive by means of mRDT and 4.3% (6/138) with a negative TBS were tested positive by means of mRDT. In other words, 94.1% (32/34) of paediatric inpatients with a positive TBS received a negative mRDT while 95.7% (132/138) of paediatric inpatients with a negative TBS received a negative mRDT result.

**Table 4:** Malaria RDT vs. TBS results of medical adult and paediatric inpatients where both mRDT and TBS were performed.

	Total	Adult medical inpatients				Pediatric medical inpatients			
		Total	TBS positive N = 11	TBS negative N = 56	p-value	Total	TBS positive N = 34	TBS negative N = 138	p-value
Total Number of Patients–n/N (%)	239	67	11/67 (16.4)	56/67 (83.6)		172	34/172 (19.8)	138/172 (80.2)	
mRDT positive–n/N (%)	13/239 (5.4)	5/67 (7.5)	3/11 (27.3)	2/56 (3.6)	0.006	8/172 (4.7)	2/34 (5.9)	6/138 (4.3)	0.704
mRDT negative–n/N (%)	226/239 (94.6)	62/67 (92.5)	8/11 (72.7)	54/56 (96.4)	0.006	164/172 (95.3)	32/34 (94.1)	132/138 (95.7)	0.704

**Fig 5:**
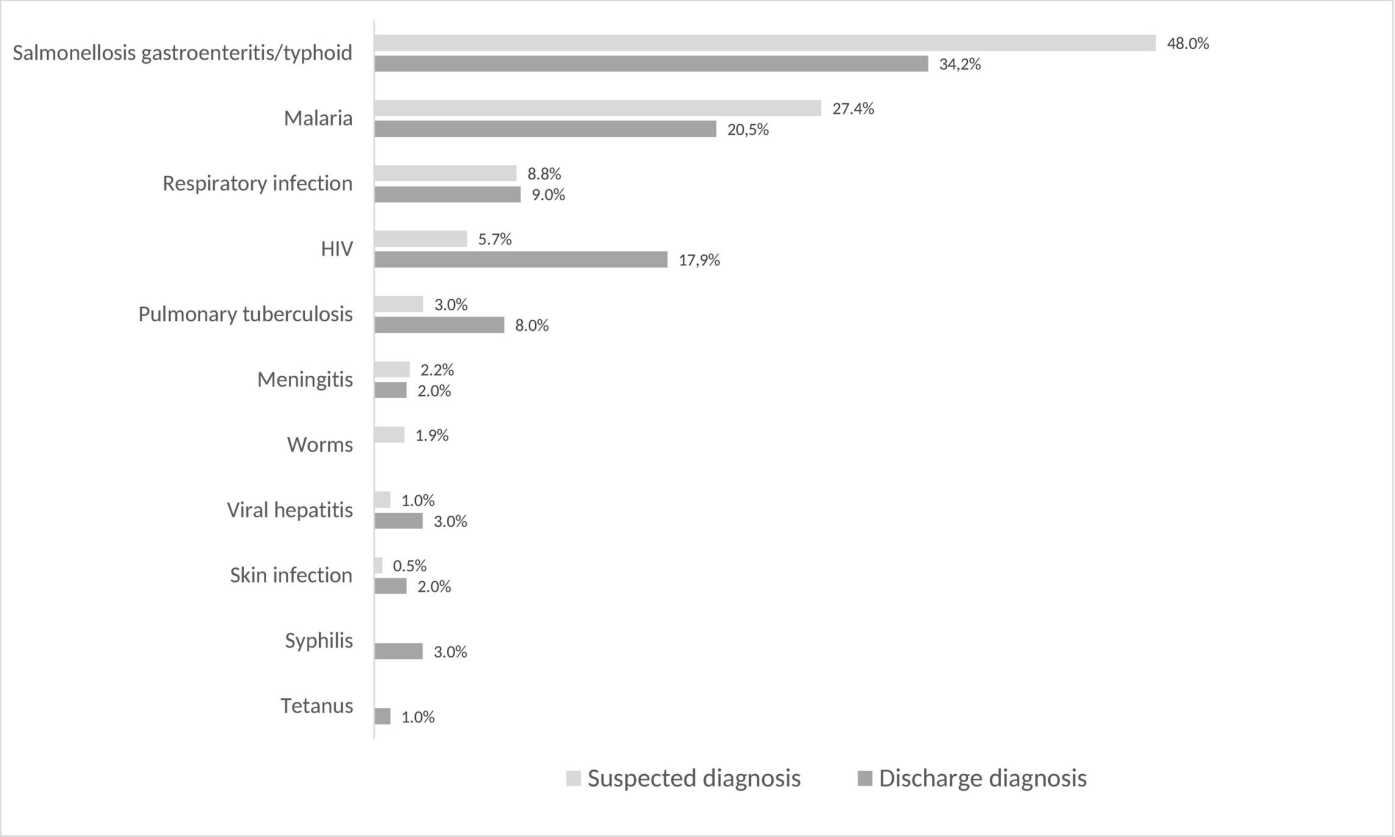
Proportions of diagnosed infectious diseases in medical adult patients.

**Fig 6:**
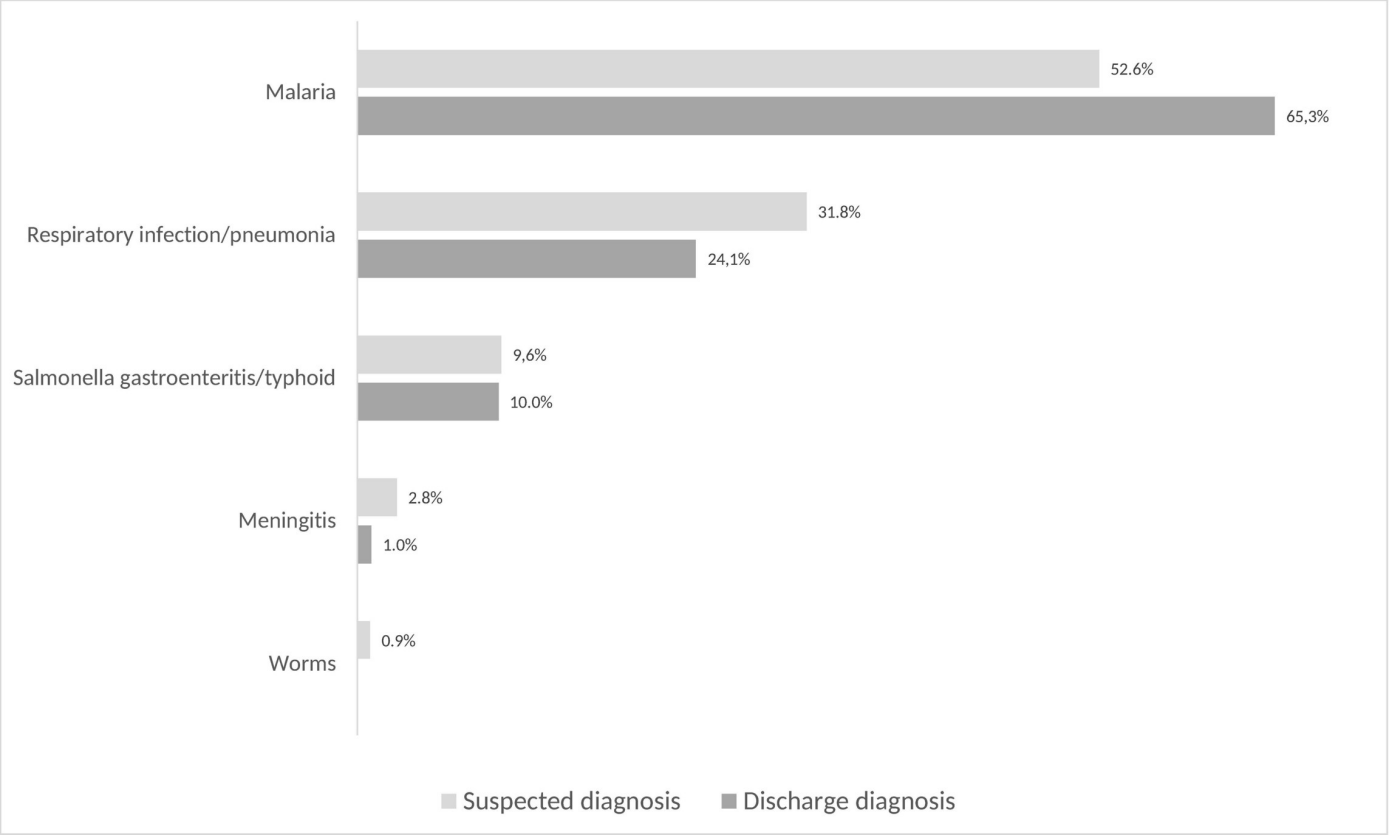
Proportions of diagnosed infectious diseases in medical paediatric patients.

Overall, the most frequent discharge diagnoses of inpatients were malaria (31.3%; 222/710), respiratory infection/pneumonia (11.7%; 83/710) and salmonella gastroenteritis/typhoid (9.7%; 69/710). Within the diagnosis group infectious diseases, most common discharge diagnoses for adult inpatients were salmonella gastroenteritis/typhoid (34.2%; 40/117), malaria (20.5%; 24/117) and HIV (17.9%; 21/117). Particularly HIV was posed more frequently as discharge diagnosis than as suspected diagnosis (Fig 5). For paediatric patients, the most frequent discharge diagnoses within the diagnosis group infectious diseases were malaria (65.3%; 198/303), respiratory infection/pneumonia (24.1%; 73/303) and salmonella gastroenteritis/typhoid (9.6%; 29/303). Within this group, particularly malaria was posed more frequently as discharge diagnosis than as suspected diagnosis (Fig 6).

Altogether, malaria was diagnosed significantly more often compared to other diagnoses when mRDT or TBS showed positive test results (Table 5). However, 62 of 710 patients (8.6%) received a different discharge diagnosis than malaria even though positive mRDT or TBS indicated infection with *P*. *falciparum*. Similarly, 91 of 710 patients (12.8%) received malaria as discharge diagnosis even though mRDT or TBS did not indicate infection with *P*. *falciparum*.

**Table 5:** Malaria as final diagnosis vs. malaria diagnostic test results.

	Total	Adult medical inpatients				Pediatric medical inpatients			
		Total	Diagnosis malaria N = 24	Other diagnosis N = 294	p-value	Total	Diagnosis malaria N = 198	Other diagnosis N = 194	p-value
Total Number of Patients–n/N (%)	710	318	24/318 (7.5)	294/318 (92.5)		392	198/392 (50.5)	194/392 (45.5)	
Patients with mRDT positive–n/N (%)	208/710 (29.3)	20/318 (6.3)	7/24 (29.2)	13/294 (4.4)	<0.001	188/392 (48.0)	152/198 (76.8)	36/194 (18.6)	<0.001
Patients with mRDT negative–n/N (%)	350/710 (49.3)	148/318 (46.5)	16/24 (66.7)	132/294 (44.9)	<0.001	202/392 (51.5)	44/198 (22.2)	158/194 (81.4)	<0.001
Patients with TBS positive–n/N (%)	48/710 (6.8)	14/318 (4.4)	6/24 (25.0)	8/294 (2.7)	<0.001	34/392 (8.7)	29/198 (14.6)	5/194 (2.6)	<0.001
Patients with TBS negative–n/N (%)	224/710 (31.5)	85/318 (26.7)	8/24 (33.3)	77/294 (26.2)	<0.001	139/392 (35.5)	7/198 (3.5)	132/194 (68.0)	<0.001

### Inpatient expenses

We calculated average inpatient healthcare expenses for all adult and paediatric inpatients based on laboratory expenses, expenses for medications and hospitalization costs (Table 6). Average laboratory expenses per adult inpatient were 51,700 Guinean Francs (GNF; 9000 GNF = approximately 1 USD), for medications GNF 35,9000 and hospitalization costs GNF 85,000, amounting to average healthcare expenses per adult inpatient of GNF 49,5700 or around 207 purchasing power parity (PPP) for actual health (2400 GNF = approximately 1 PPP actual health expenditure). Average laboratory expenses per paediatric inpatient were GNF 19,700, for medications GNF 229,000 and hospitalization costs GNF 80,000, amounting to average healthcare expenses per inpatient of GNF 328,700 or roughly 137 PPP for actual health.

**Table 6:** Inpatient healthcare expenses.

	Medical adult inpatients	Medical paediatric inpatients
**Medications**		
Average price per medication (excluding 10 most expensive drugs)	51,700 GNF	51,700 GNF
Total medications taken by patients	2210	1735
Total number of patients	318	392
Average medications per patient	7	4
Total cost for medications—in GNF	114,257,000 GNF	89,699,500 GNF
Average cost for medications per patient—in GNF	359,000 GNF	229,000 GNF
**Other**		
Emergency room fee—in GNF	15,000 GNF	10,000 GNF
Hospitalization fee—in GNF	70,000 GNF	70,000 GNF
**Totals**		
Laboratory costs per patient—in GNF	51,700 GNF	19,700 GNF
Medication costs per patient—in GNF	359,000 GNF	229,000 GNF
Hospitalization costs per patient—in GNF	85,000 GNF	80,000 GNF
Total costs per patient—in GNF	495,700 GNF	328,700 GNF
Total costs per patient—in PPP	207	143
Average GNI per capita in Guinea (2018)—in USD	$850	$850
Total inpatient cost per patient—in % of GNI	6.5%	4.4%
Average per capita spending on healthcare in Guinea (2017)—in USD	$34	$34
Average per capita spending on healthcare in Guinea (2017)—in % of GNI	4.1%	4.1%

## Discussion

With this study we hope to provide more insight into the local epidemiology of febrile illness in south-eastern Guinea, a region at high risk for outbreaks of diseases with epidemic potential such as Ebola virus disease. We described the clinical and diagnostic characteristics, treatments and outcomes of patients with febrile illness and compared them to non-febrile patients. Based on our findings, we can point to some management needs regarding diagnostic practices and treatments of febrile illness, side-lined by an estimation of the economic burden for patients seeking healthcare.

Febrile illness is a frequent reason for patients seeking healthcare in south-eastern Guinea. During our study period, the typical febrile patient came to the emergency room with asthenia, loss of appetite, headache, cough and abdominal pain. Together with fever, these symptoms guided clinicians towards suspecting the most common diseases in the region: malaria, diarrheal diseases/gastroenteritis and respiratory infection [34].

### Malaria

Since malaria (*P*. *falciparum*) is endemic in south-eastern Guinea, it is evidently assumed to be the most common cause for febrile illness [35]. Ruling out malaria in febrile patients is a primary task for emergency medicine in the region [6, 10]. While mRDT are commonly used for this task, our study shows that mRDT test results do not necessarily correspond to malaria as suspected or discharge diagnoses. Furthermore, a large proportion of mRDT test results do not correspond to TBS results. In our study, 12.8% of all patients received malaria as discharge diagnosis despite negative diagnostic test results. Thus, point-of-care and laboratory diagnostic procedures seem to be only partly relevant when diagnosing malaria in south-eastern Guinea. Studies have indicated that mRDT and TBS in field settings miss roughly between 10–20% of malaria infections [36, 37]. This means that clinical experience is an important factor when diagnosing malaria. However, it has been pointed out that the practice of misdiagnosing malaria–due to malaria being the most ready-at-hand diagnosis for febrile illness–plays an equally important role in low-resource African healthcare settings and leads to an over-consumption of resources as well as more antimalarial drug resistance development [38, 39]. Since our study is only descriptive in regard to local diagnostic practices of febrile illness, we cannot ascertain true or false positive malaria cases. We can only emphasize that in malaria endemic regions at high risk for outbreaks of less common but highly infectious diseases with epidemic potential, investigation into the causes for febrile illness should at times go beyond the most probable entity, here malaria. This is in agreement with the Guinean national guidelines regarding malaria: when mRDT or TBS rule out malaria as the cause for febrile illness, other causes should be considered and investigated [40]. Furthermore, the assumed index case of the 2021 Ebola outbreak in south-eastern Guinea went unrecognized and was reportedly only treated against malaria by a number of different clinics as mRDT indicated infection with *P*. *falciparum*. Already the 2014–2016 West African Ebola virus epidemic has produced studies highlighting the relevance of malaria-sensitive screening tools for non-malarial illness such as Ebola virus disease and cases with coinfection [41, 42].

### Salmonella gastroenteritis

Besides malaria, salmonella gastroenteritis/typhoid as diarrheal disease is another common diagnosis in south-eastern Guinea in patients with febrile illness, especially in adults. Widal TO/TH agglutination test is widely used and plays a major role in ascertaining this diagnosis. While this low-cost point-of-care test may be indicative of enteric fever in certain clinical situations and may be used according to Guinean national guidelines, its use is often discouraged due to its low specificity [43, 44]. In our study, 18.2% of all inpatients had a positive Widal TO/TH test result and a considerable proportion of patients was treated with antimicrobial drugs commonly used for gastroenteritis, namely ampicillin, metronidazole and ceftriaxone. A false diagnosis of enteric fever through Widal TO/TH agglutination test may result in the unnecessary use of antimicrobial drugs and provoke the development of drug-resistant bacteria [45, 46]. Moreover, it may lead to the non-consideration of diseases with epidemic potential as the cause for febrile illness [47]. Further strengthening of diagnostic capacities, particularly the possibility to perform blood cultures and antibiograms would undoubtedly improve identification and treatment of febrile illnesses caused by common gastrointestinal and systemic diseases.

### Respiratory infection/pneumonia

The third most important infectious disease entity frequently assumed in febrile patients is respiratory infection, especially in children. Due to the high fees and therefore low use of radiological imaging (x-ray), calculated antimicrobial treatment is the default option for suspected respiratory infection/pneumonia. Other diagnostic tests for respiratory infections are only used sporadically. Our study shows that a large majority of patients (58.1%) is treated with ampicillin and only rarely with amoxicillin. National guidelines recommend the use of amoxicillin for the treatment of respiratory infections. One reason for the observed preference of ampicillin over amoxicillin might be the fact that it can be administered intravenously, which is the case for its use at the HRNZ, as it is the case for the use of paracetamol at the HRNZ as well. Social scientific research on vaccination in Guinea has shown that Guineans favour intravenous application of therapeutic agents because local understandings of medical therapies attribute a higher power and efficacy to injections over orally administered medications [48]. However, excessive use of these antimicrobial agents against respiratory infections may lead to drug resistance [49, 50]. In addition, unnecessary parenteral application of antibiotics and other medications bears higher risks of complications such as venous catheter infections. We believe that Guinean healthcare structures could highly benefit from antimicrobial stewardship programs specifically designed for low-resource settings [51].

Further, at the time of writing (July 2021), more than 23000 COVID-19 cases have been reported in Guinea [52]. RT-PCR testing for SARS-CoV-2 is currently being expanded in Forest Guinea [53]. Studies on the knowledge, attitudes and practices of Guinean healthcare workers towards COVID-19 show that knowledge, awareness and infection prevention and control practices regarding COVID-19 may still be improved through regular trainings and interventions [54]. However, it is still unclear how COVID-19 will be handled in the long-term clinical routine in low-resource settings. For Guinea, we think that COVID-19 should be integrated as a potential differential diagnosis for febrile illness and respiratory infection alongside more common diseases without giving it priority over others or side-lining more relevant causes for respiratory infection. The nationally reported total death toll for COVID-19 in Guinea since March 2020 is still considerably low (n = 353) compared to the toll respiratory infections/pneumonia take on the general population and especially children [55].

### HIV, tuberculosis, meningitis viral haemorrhagic fevers

We showed that HIV, tuberculosis and meningitis are possibly amongst leading causes for febrile illness in Forest Guinea. Furthermore, viral haemorrhagic fevers, especially Ebola virus disease will remain important differential diagnoses in the region, considering the new role survivors play in sparking regional outbreaks years after recovery [56, 57]. HIV and tuberculosis in the region are mainly diagnosed and managed through internationally funded and coordinated programs. As mentioned, suspect HIV and Tuberculosis patients in the hospital setting are screened through antibody-antigen testing (HIV) or clinical presentation (tuberculosis) and usually referred to the corresponding programs for further diagnosis and treatment.

Bacterial meningitis is diagnosed at the HRZN through cerebrospinal fluid examination and is usually treated with antimicrobial drugs. However, isolation of suspect and confirmed cases is uncommon. Furthermore, viral meningitis can neither be diagnosed due to missing PCR capacities nor properly treated due to the high costs of antiviral medications such as Aciclovir. Every meningitis is treated as bacterial meningitis. Moreover, the high use of antibiotics at the level of the emergency room risks to render cerebrospinal fluid diagnostics less effective. We thus reiterate our call for antimicrobial stewardship while hoping that international and public funds made available to the Guinean healthcare system due to Ebola and COVID-19 will be able to address issues of lacking diagnostics and treatments for meningitis.

Viral hemorrhagic fever diagnostic capacities and trainings were scaled up in Forest Guinea, particularly after the resurgence of Ebola in February 2021. As a consequence, some facilities in the region are currently undergoing fundamental changes in terms of triaging and screening febrile patients. It seems that viral hemorrhagic fevers are now being taken seriously [58]. While these steps are doubtlessly important, it has been argued that establishing trust between patients and healthcare workers in the case of viral hemorrhagic fever screening is as important as an adequate biomedical response [59, 60].

### Inpatient mortality and healthcare expenses

During our study period, overall mortality of medical inpatients was fairly high (18.0%) and even higher for febrile patients in comparison to non-febrile patients. Inpatient mortality in West African hospitals are roughly between 5–25% even though comparison of mortality rates is difficult due do different reporting practices and varying mortality rates in different services [61–64]. Nevertheless, our reported inpatient mortality rate is considerable and speaks to a wide array of interconnected issues such as patient morbidity, underlying factors regarding health-seeking behaviour and the quality of healthcare in some West African countries [65]. One particular reason for the delayed treatment of patients–a factor causing increased mortality for certain diseases–is economic constraint coupled with high out-of-pocket user fees in proportion to income [66–68]. We calculated that an adult inpatient spends roughly around 6.5% (in children 4.4%) of the per capita gross national income in Guinea as in 2018 [69]. This measures up to an enormous economic burden on inpatients and their families. Structural improvements in healthcare such as enhanced diagnostic capacities and advances in treatments for certain diseases as currently envisaged in post-Ebola Guinea must go hand in hand with propositions for better access [70].

### Limitations of the study

The first limitation of our study is that we only collected patient data at one particular hospital. This reduces generalizability of our findings. However, the epidemiology, the socio-economic conditions and healthcare provision in the little-accessible border region of south-eastern Guinea, Sierra Leone and Liberia are fairly similar and we thus believe that our findings are very important for this high-risk zone for outbreaks of diseases with epidemic potential [71].

Second, a study period of three months is relatively short. Our data does not capture seasonal disease patterns potentially causing a changing epidemiology of febrile illness. Data was collected during the dry season where transmission of malaria is usually lower than in the wet season, meaning that the average annual proportion of diagnosed malaria and positive malaria tests may actually be higher than reported in our study [72].

Third, our study was not designed to verify the aetiology of febrile illness in patients. Its aim was simply to describe the local epidemiology of febrile illness as it is produced in a low-resource setting. Despite its disadvantages, we believe that this approach increases understanding of local clinical practices and highlights potential sites for improvement.

Fourth, our calculation regarding average inpatient expenses rely on a rough estimation on fees for medicines. We were only able to review the amount of medications each inpatient used but not the specific medications themselves. We adjusted average prices for medicines by eliminating the ten most expansive medications from our calculations to reduce skewing and hence risk of over-reporting medication fees. Nevertheless, our calculations remain rough estimates and should only be understood as a means to underscore the high economic burden of inpatient care.

## Conclusions

Our study highlights the importance of malaria, salmonella gastroenteritis/typhoid and respiratory infection in patients with febrile illness in south-eastern Guinea. These diseases are mainly diagnosed on clinical grounds and rapid point-of-care diagnostic tests. Common serological and other diagnostic measures to ascertain aetiology of febrile illness are rarely used or only partially regarded. This practice risks to miss signal cases of highly infectious diseases such as Ebola virus disease. Diagnostic capacities in regions at risk for diseases with epidemic potential, such as south-eastern Guinea, should be enhanced. Furthermore, antimicrobial medications play a major role in treating febrile illness, increasing the possibility for drug resistance. Guinean hospitals would benefit from antimicrobial stewardship. Patients with febrile illness have a high inpatient mortality rate and the economic burden of inpatient care on patients and families is considerable. This underscores the importance of linking structural improvements in healthcare provision to increased access to healthcare.

## Supporting information

S1 Table (PDF)
