# Supplementary material for: Clinical presentations, diagnostics, treatments and treatment costs of children and adults with febrile illness in a tertiary referral hospital in south-eastern Guinea: A retrospective longitudinal cohort study
Source: PLoS One. 2022 Jan 10;17(1):e0262084. doi: 10.1371/journal.pone.0262084 (PMC8746772; doi:10.1371/journal.pone.0262084)
Supplement: S1 Table — (PDF) [file pone.0262084.s001.pdf]

| Infectious disease | Diagnostic test | Comment |
|---|---|---|
| Malaria | Malaria rapid diagnostic test (mRDT), Thick blood smear (TBS) | Malaria in Guinea is presumably caused by *P. falciparum* only. Diagnosis is based on mRDT and/or positive TBS, or clinical presentation only. |
| Gastroenteritis/ Typhoid fever | Widal TO/TH | A positive Widal test and corresponding clinical symptoms play a crucial role in diagnosing gastroenteritis/typhoid fever in Guinea. Blood cultures were unavailable during time of study. Typhoid fever and salmonella gastroenteritis are not distinguished in the Guinean clinical context. |
| Respiratory infection | X-ray | Diagnosis based on clinical presentation and occasionally on radiological results; diagnosis includes all respiratory infections other than tuberculosis. |
| Liver disease | Sonography, Hepatitis B surface antigen | Diagnosis based on clinical presentation, sonography and/or positive Hepatitis B surface antigen results. |
| Meningitis | Cerebrospinal fluid | Cerebrospinal fluid is used to diagnose bacterial meningitis only. PCR testing for viral meningitis was unavailable during time of study. Diagnosis based on clinical presentation and/or cerebrospinal fluid results. |
| HIV | HIV antibody-antigen test | HIV antibody-antigen tests are used to diagnose HIV. If positive, patients are referred to the HIV clinic for further PCR testing. |
| Pulmonary tuberculosis | Sputum, X-ray | Pulmonary tuberculosis is usually diagnosed and treated by the external tuberculosis clinic. Hospital diagnosis is based on clinical presentation and/or radiological findings and/or sputum analysis. |
| Parasitic infection | Stool microscopy parasites | This is the only diagnostic test available for recognizing common parasitic infection . |
| Syphilis | TPHA | Only TPHA screening test is used, confirmation test was unavailable during time of study. |
| Toxoplasmosis | IgG/IgM | Direct detection of Toxoplasmosis was unavailable during time of study. |
